# An In Vitro RNA Editing-Based Reporter Assay for Transcriptional Activity of Therapeutic Gene in Gene Therapy Products

**DOI:** 10.3390/molecules29225312

**Published:** 2024-11-11

**Authors:** Lei Yu, Yong Zhou, Guangyu Wang, Jianning Fu, Zhihao Fu, Chenggang Liang, Junzhi Wang

**Affiliations:** 1 School of Life Science and Biopharmaceutics, Shenyang Pharmaceutical University, No. 103 Wenhua Road, Shenyang 110016, China; yulei@nifdc.org.cn (L.Y.); 15261391872@163.com (J.F.); 2 State Key Laboratory of Drug Regulatory Science, National Institutes for Food and Drug Control, No. 31 Huatuo St., Daxing District, Beijing 100050, China; zhouyong@nifdc.org.cn (Y.Z.); wanggy@nifdc.org.cn (G.W.); fuzhihao@nifdc.org.cn (Z.F.)

**Keywords:** RNA editing, adenosine deaminases acting on RNA, transcriptional activity, gene therapy products, reporter gene assay, recombinant adeno-associated virus

## Abstract

The expression of therapeutic genes is critical for the efficacy of gene therapy products. However, existing methods such as immunological analysis at the protein level or reverse-transcription PCR at the RNA level are unable to accurately quantify the expression activity of the target gene. Herein, an in vitro RNA editing-based reporter assay was developed to detect specific mRNA. The designed sensor RNA could specifically identify the target mRNA, and the reporter gene was activated in a dose-dependent manner because of RNA editing mediated by endogenous adenosine deaminases acting on RNA. Of note, all sensors that targeted different regions, including the gene of interest, tag sequence, and 3′ untranslated region, showed a dose-dependent response pattern. The sensor reporter assay, which was used for quantifying the transcriptional activity of recombinant adeno-associated virus-based gene therapy products, revealed excellent performance in terms of assay specificity, precision (inter-assay relative standard deviation < 15%), accuracy (90–115% recovery), and linearity (R^2^ > 0.99). The reporter assay could also be employed for other gene therapy vectors, including mRNA and recombinant lentivirus. Thus, a robust and reliable platform was developed for assessing the transcriptional activity of therapeutic genes, thereby offering a powerful tool for the quality control of gene therapy products.

## 1. Introduction

Gene therapy in humans modifies or manipulates the expression of genes or alters the biological properties of living cells for therapeutic purposes. It functions in different ways: replacing a disease-causing gene with a healthy form, inactivating a malfunctioning disease-causing gene, or inserting a new or modified gene into the genome to combat a disease [1,2]. Effective delivery systems, including viral and nonviral vectors, are essential for the delivery of therapeutic sequences [3,4,5,6]. Unlike traditional medications, the potency of gene therapeutics depends on multiple factors. For example, the efficacy of recombinant adeno-associated virus (rAAV)-based gene therapy products depends on capsid infectivity, therapeutic gene expression, and, ultimately, protein function [7]. The expression level of the intended therapeutic gene is typically assessed using methods such as Western blot, enzyme-linked immunosorbent assay, or immunostaining after the gene has been delivered into the test cells [8,9,10,11]. In general, therapeutic genes are functional genes that are commonly expressed across various cell lines, which necessitates the utilization of cells with gene knockout to minimize background interference [12]. To increase expression or bioactivity, the coding sequences of most gene therapy products are functionally optimized, in which the transcribed mRNA sequences significantly differ from the original sequence but still encode identical amino acids [13]. This makes mRNA a better indicator for assessing therapeutic gene expression. However, mRNA detection relies on RNA extraction and reverse-transcription polymerase chain reaction (RT-PCR), which are tedious and susceptible to ubiquitous RNases [14].

Adenosine deaminases acting on RNA (ADARs) are RNA-editing enzymes that convert adenosines (A) to inosines (I) in structured and double-stranded RNAs [15,16,17,18,19]. Most cellular machinery read I as guanosines (G), resulting in A-to-G editing [15]. In mammals, three ADAR family members have been identified: ADAR1 and ADAR2 exhibit catalytic activity, whereas ADAR3 appears to be inactive [20,21]. During A-to-G editing at the TAG, TGA, or TAA termination codon, the termination codon is converted to the TGG (Trp) codon. This property of ADARs has been exploited for treating diseases caused by nonsense mutations [22]. In addition, ADAR-based RNA sensors have been developed to detect specific RNA sequences in living cells, offering new tools for cell monitoring and gene regulation [22,23]. However, there is no report on their use in the activity analysis of gene therapy products.

**Figure 1:**
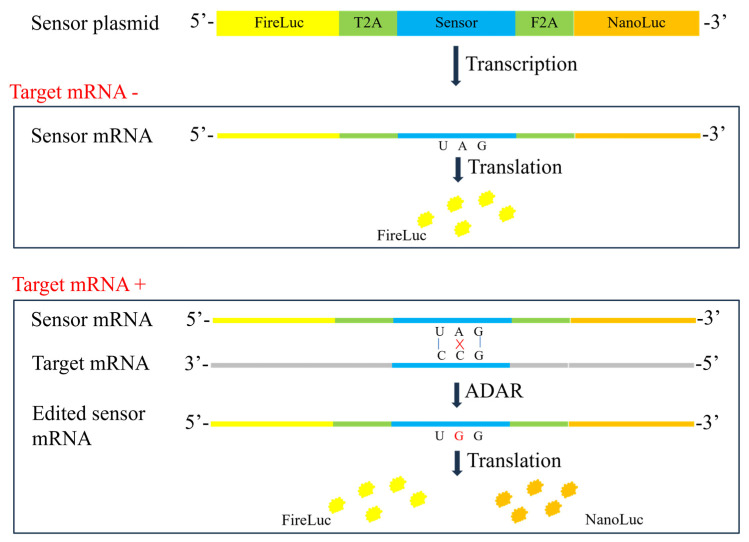
RNA editing-based reporter assay for detecting target mRNA. FireLuc, firefly luciferase; NanoLuc, nano-luciferase; T2A, *Thosea asigna* virus 2A; F2A, foot-and-mouth disease virus 2A.

In the present study, an in vitro RNA editing-based reporter assay was developed to assess the transcriptional activity of the therapeutic gene in gene therapy products such as AAV. To develop this assay, a target sequence containing a tandem CCA was initially identified in the therapeutic gene. Next, a sensor sequence complementary to the target sequence with an edit-enhancing C:A mismatch at a central TAG stop codon was constructed and inserted between an upstream reporter and a downstream reporter. The constitutively expressed upstream reporter was designed to act as an internal reference. In the sensor sequence, the central stop codon (UAG) prevents the translation of the downstream reporter, thereby ensuring the expression of only the upstream reporter. Figure 1 illustrates the principle of the RNA editing-based reporter assay for detecting target mRNA. The sensor plasmid comprises a firefly luciferase sequence (upstream reporter), *Thosea asigna* virus 2A (T2A), sensor sequence, foot-and-mouth disease virus 2A (F2A), and nano-luciferase (downstream reporter). The T2A and F2A elements are commonly employed for the coexpression of multiple proteins in a single transcript [24,25]. Sensor mRNA is transcribed from the sensor plasmid, and target mRNA is transcribed from the therapeutic gene delivered by gene therapy vectors. In the absence of target mRNA, the translation of nano-luciferase is inactivated and only firefly luciferase is translated. In the presence of target mRNA and sensor mRNA, dsRNA is formed. The dsRNA recruits ADAR to perform A-to-G editing in the UAG stop codon, enabling the translation of nano-luciferase. Sensors that target specific regions were designed and introduced into 293T cells, which were treated with serial dilutions of gene therapy vectors carrying a specific gene of interest (GOI). The results showed that the activation of the downstream reporter was positively correlated with the quantity of vectors introduced. Moreover, different gene therapy vectors, including plasmid, AAV, mRNA, and lentivirus, exhibited a dose-dependent response in the developed mRNA reporter assay. The reporter assay was used to determine the transcriptional activity of rAAV products, and the performance of the assay was assessed through preliminary validation.

## 2. Results

### 2.1. Construction of Sensors for Specific mRNA

To facilitate assay development, the pAAV-GFP-WPRE plasmid containing a green fluorescence protein (GFP) sequence and a woodchuck hepatitis virus post-transcriptional regulatory element (WPRE) in the 3′ untranslated region (3′ UTR) was used. Homo sapiens survival of motor neuron 1 (SMN1, NM_000344.4) gene was inserted into the upstream of the GFP sequence to construct the pAAV-SMN-GFP-WPRE plasmid. Sensors targeting GOI (i.e., SMN), tag sequence (GFP), or 3′ UTR (WPRE) were designed and inserted into the double-reporter vector. Figure 1 illustrates the construction of the double-reporter vector, and the sequences of sensors and corresponding target sites are detailed in Section 4.2.

#### 2.1.1. Response of Sensors Targeting Different Regions

**Figure 2:**
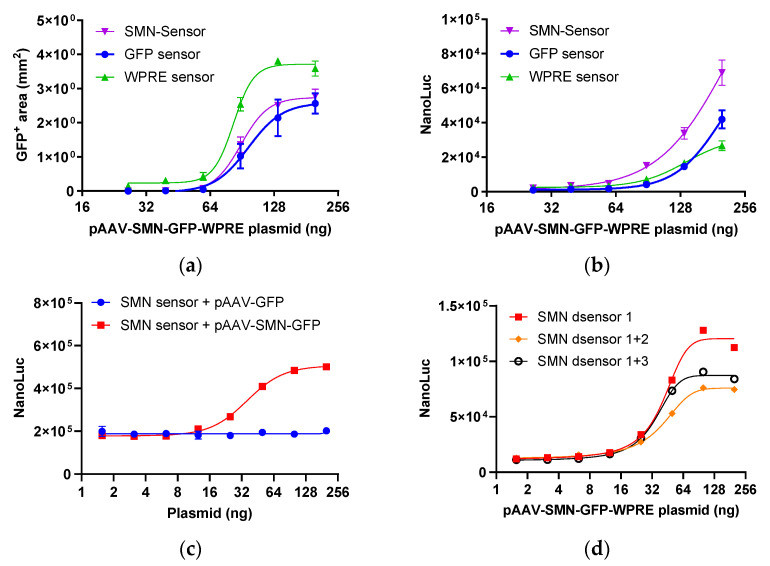
Response of sensors targeting different regions. (**a**) GFP expression of 293T cells cotransfected with sensor reporters and pAAV-SMN-GFP-WPRE. (**b**) Response of the GFP sensor, WPRE sensor, and SMN sensor to pAAV-SMN-GFP-WPRE. (**c**) Response of the SMN sensor to pAAV-GFP and pAAV-SMN-GFP-WPRE. (**d**) Response of the SMN sensor with double termination codons to pAAV-SMN-GFP-WPRE. Each plot represents two replicates. NanoLuc, nano-luciferase.

To assess the efficacy of the different sensors, they were cotransfected with various dilutions of the pAAV-SMN-GFP-WPRE plasmid into 293T cells. The expression level of GFP and nano-luciferase was measured 48 h after transfection. The expression was visualized using a live cell imaging system, and the proportion of GFP-positive cells was quantified. As shown in Figure 2a, the GFP-positive area increased in a dose-dependent manner. Likewise, the SMN, GFP, and WPRE sensor responses correlated positively with the plasmid concentration, with the SMN sensor exhibiting the strongest response, followed by the GFP sensor and the WPRE sensor (Figure 2b). These results suggest that endogenous ADARs in 293T cells effectively mediate A-to-G editing and that the sensor reporters are proficient at assessing the transcriptional activity of the target gene. Next, stably transfected reporter 293T cells were established for use in subsequent assays. When the SMN sensor 293T cells were transfected with serial dilutions of the pAAV-GFP-WPRE or pAAV-SMN-GFP-WPRE plasmid, a dose-dependent response was observed only with pAAV-SMN-GFP-WPRE, indicating that the SMN sensor is highly specific (Figure 2c).

#### 2.1.2. SMN Sensors with Double Termination Codons

Stably transfected sensor reporter cells exhibited a significantly high background of nano-luciferase activity (Figure 2c), indicating remarkable translational readthrough. To enhance the efficacy of translation termination and reduce background luciferase activity, the sensor sequence was engineered to incorporate two stop codons. SMN dsensor 1 featured a single stop codon and was used as a control. Meanwhile, SMN dsensor 1 + 2 had two consecutive stop codons and SMN dsensor 1 + 3 comprised two stop codons separated by a 15-nucleotide sequence. As depicted in Figure 2d, the nano-luciferase background activity of dsensor 1 + 2 and dsensor 1 + 3 was comparable to that of dsensor1. This finding suggests that additional mechanisms are at play in the basal expression of nano-luciferase. The incorporation of two stop codons reduced the increase in nano-luciferase activity, as the translation of the downstream nano-luciferase can only be triggered by simultaneous editing at both stop codons.

### 2.2. Effect of Overexpressed ADARs

**Figure 3:**
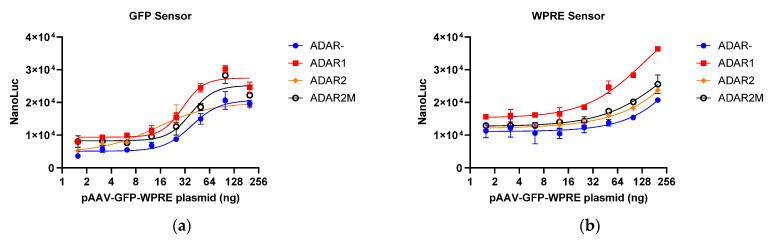
Effect of different ADARs on the reporter assay. (**a**) Response of the GFP sensor to pAAV-GFP-WPRE. (**b**) Response of the WPRE sensor to pAAV-GFP-WPRE. ADAR2M, mutated ADAR2 (E488Q). NanoLuc, nano-luciferase.

It has been reported that ADAR protein overexpression can promote A-to-G editing efficiency [15]. Three different ADAR proteins, viz. ADAR1, ADAR2, and a hyperactive mutant ADAR2 (E488Q), were introduced into stably transfected sensor cells to assess whether they can improve the sensor response. As shown in Figure 3a,b, both GFP and WPRE sensors exhibited a dose-dependent response pattern with the pAAV-GFP-WPRE plasmid. Although ADAR protein overexpression resulted in increased A-to-G RNA editing, background nano-luciferase activity also increased, resulting in an overall unchanged signal-to-noise ratio (SNR). These data suggest that ADAR protein overexpression is not necessary for optimal reporter assay results.

### 2.3. Response of the Reporter Assay to Different Gene Therapy Vectors

**Figure 4:**
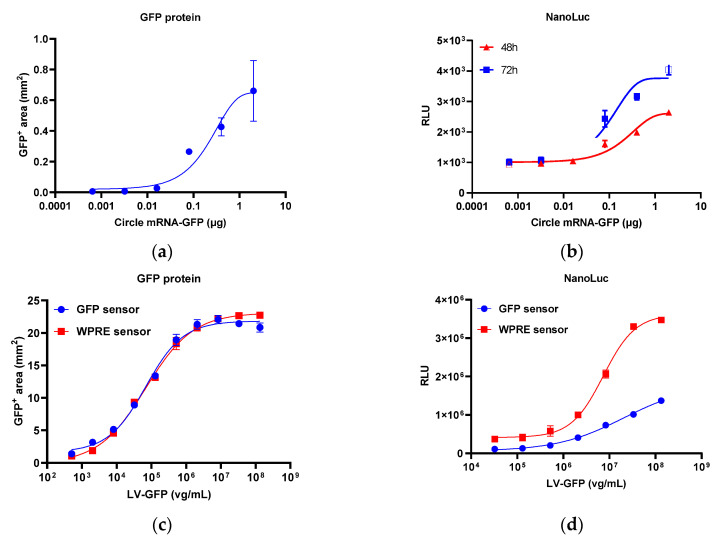

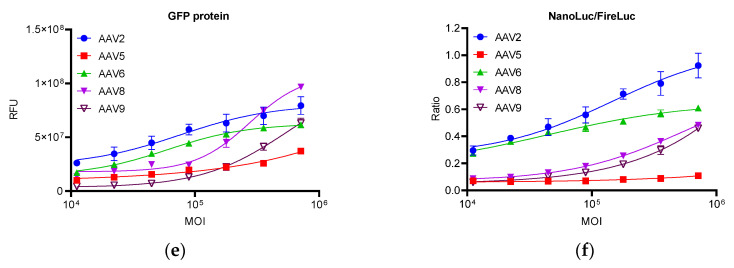
Dose–response relationship of circle mRNA, lentivirus, and different AAVs. (**a**) GFP protein expression of circle mRNA. (**b**) Nano-luciferase activity of circle mRNA. (**c**) GFP protein expression of lentivirus. (**d**) Nano-luciferase activity of lentivirus. (**e**) GFP protein expression of different AAVs. (**f**) Nano-luciferase activity of different AAVs. Each plot represents two replicates. FireLuc, firefly luciferase; NanoLuc, nano-luciferase.

In addition to the plasmid, other gene therapy vectors harboring the GFP sequence, including circle mRNA, lentivirus, and AAV, were assessed using GFP sensor reporter 293T cells. All samples exhibited dose-dependent GFP expression (Figure 4a,c,e). For detecting mRNA, the response of the GFP sensor to circle GFP mRNA samples was analyzed, revealing a dose-dependent response of nano-luciferase, which was similar to the expression of GFP (Figure 4a,b). For lentivirus, lentiviruses carrying GFP and WPRE sequences were analyzed using the GFP sensor and WPRE sensor reporter 293T cells, respectively. As shown in Figure 4c,d, in both sensor reporter cells, the response of nano-luciferase was consistent with the expression of GFP, with the WPRE sensor exhibiting a higher response than the GFP sensor. As a ubiquitous sequence in the lentivirus vector, the WPRE sensor reporter cell can be used as a universal test cell for lentivirus samples containing WPRE sequences. In the AAV assay, different AAV vector serotypes, including AAV2, 5, 6, 8, and 9, exhibited a dose-dependent response similar to GFP expression (Figure 4e,f).

### 2.4. Transcriptional Activity of rAAV Products Determined Using the Developed Reporter Assay

The aforementioned results suggested that the reporter assay can be used to assess the efficacy of gene therapy products. Thus, the ability of the assay to determine the transcriptional activity of rAAV products was next assessed.

#### 2.4.1. Specificity of the Reporter Assay

**Figure 5:**
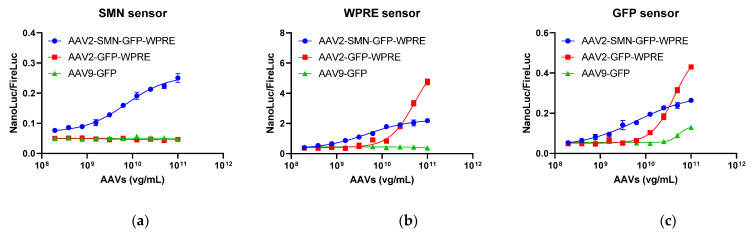
Specificity of the sensor reporter assay. (**a**) Response of the SMN sensor. (**b**) Response of the WPRE sensor. (**c**) Response of the GFP sensor. Each plot represents two replicates. FireLuc, firefly luciferase; NanoLuc, nano-luciferase.

To assess the specificity of the reporter assay, AAV samples harboring different elements were evaluated using SMN, GFP, and WPRE sensor reporter 293T cells. The SMN sensor demonstrated a dose-dependent response to AAV2-SMN-GFP-WPRE, whereas no response was elicited against AAV2-GFP-WPRE and AAV9-GFP (Figure 5a). Meanwhile, the WPRE sensor demonstrated a dose-dependent response to both AAV2-SMN-GFP-WPRE and AAV2-GFP-WPRE but not to AAV9-GFP (Figure 5b). Finally, the GFP sensor exhibited a dose-dependent response to all three AAVs (Figure 5c). AAV2-SMN-GFP-WPRE elicited similar dose–response patterns in the three sensor reporter cells. Likewise, AAV2-GFP-WPRE showed similar dose–response patterns in GFP and WPRE sensor reporter cells. Thus, the reporter assay was highly specific to its target sequence. In addition, sensors targeting different regions were consistent in their dose–response patterns for the same test sample.

#### 2.4.2. Relative Quantitative Method

**Figure 6:**
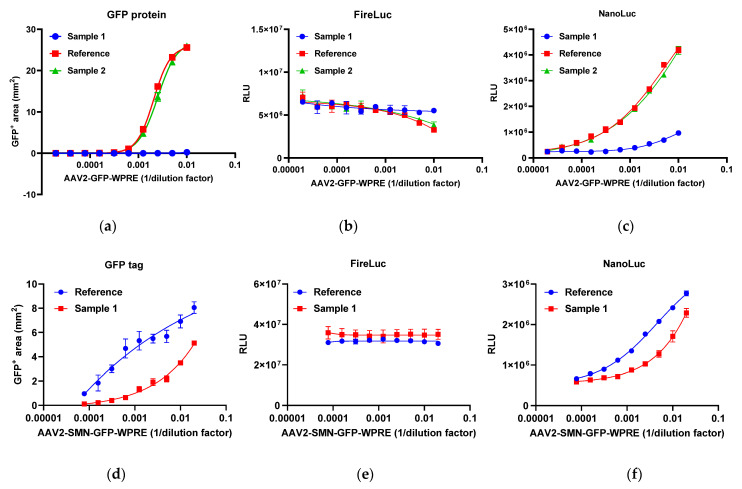

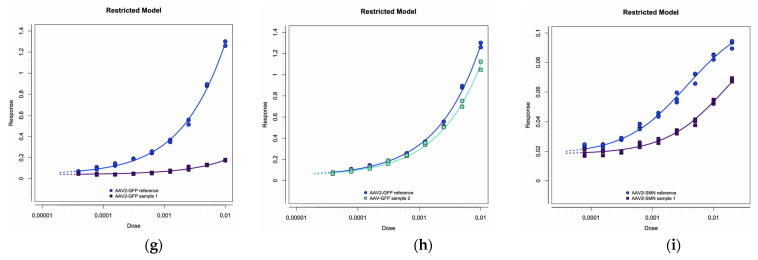
Dose–response curves of AAV2-GFP and AAV2-SMN-GFP. (**a**–**c**) GFP protein expression, firefly luciferase activity, and nano-luciferase activity of GFP sensor 293T cells treated with serial dilutions of AAV2-GFP. (**d**–**f**) GFP protein expression, firefly luciferase activity, and nano-luciferase activity of SMN sensor 293T cells treated with serial dilutions of AAV2-SMN-GFP. (**g**–**i**) Restricted 4-PL model of AAV reference and samples. Each plot represents two replicates for AAV2-GFP and three replicates for AAV2-SMN-GFP. FireLuc, firefly luciferase; NanoLuc, nano-luciferase.

The AAV2-GFP-WPRE reference and two AAV2-GFP-WPRE samples (samples 1 and 2) were analyzed using GFP sensor reporter 293T cells. Figure 6a–c show the response of GFP expression, firefly luciferase, and nano-luciferase, respectively. High-titer AAVs resulted in reduced firefly luciferase activity, indicating their negative impact on cell function. To address this issue, the nano-luciferase-to-firefly luciferase ratio was employed as the response in a four-parameter logistic (4-PL) parallel analysis. Figure 6g,h illustrate the restricted 4-PL model employing the nano-luciferase-to-firefly luciferase ratio as the response and the 1/dilution factor as the dose. The AAV2-SMN-GFP-WPRE reference and sample 1 were analyzed using SMN sensor reporter 293T cells. Figure 6d–f show the response of GFP expression, firefly luciferase, and nano-luciferase, respectively. Figure 6i shows the restricted 4-PL model. According to the *Chinese Pharmacopoeia*, F-tests were performed to evaluate the data set for any notable nonlinearity or nonparallelism at the significance level of *p* = 0.05. The potency of the reference was set to 100%, and the relative potency of the test samples was calculated through comparison with the reference using 4-PL parallel analysis.

#### 2.4.3. Precision, Accuracy, and Linearity

To evaluate precision, two AAV2-GFP-WPRE samples and one AAV2-SMN-GFP-WPRE sample were assessed five times, with the relative potency calculated using the 4-PL parallel assay. The genomic titer of the reference and samples was determined using a digital PCR (dPCR) targeting the GFP sequence. As illustrated in Table 1, the relative standard deviation (RSD) values of the five estimates were <15% for all AAV samples, indicating high precision. In addition, to assess the accuracy and linearity of the assay, AAV2-GFP-WPRE and AAV2-SMN-GFP-WPRE references with the anticipated potencies of 100%, 80%, 60%, 40%, and 20% were simultaneously analyzed. As shown in Table 2, the recovery rates were within the range of 90–115%, demonstrating high accuracy. The slopes of the linear equations exhibited a value close to 1.0, with an R^2^ > 0.99, which indicated excellent linearity.

## 3. Discussion

The expression level of therapeutic genes serves as an indicator of bioactivity in the quality control of gene therapy products. However, it is often difficult to quantify the protein expression of the therapeutic gene owing to a lack of specific antibodies or high background in the test cells [12]. Although mRNA detection is more accessible, it is tedious [26,27,28]. In the present study, an RNA editing-based reporter assay was developed to detect specific mRNA. The designed sensor RNA specifically identified the target mRNA, and the reporter gene was activated in a dose-dependent manner with the help of ADAR proteins. All sensors that targeted different regions, including SMN (GOI), GFP (tag sequence), and WPRE (3′UTR), exhibited a dose-dependent pattern. The SMN and GFP sensors contained the TAG stop codon. Once the internal A was converted to I (read as G), the translation of the downstream nano-luciferase was activated. However, no satisfactory CCA sequence was found for the WPRE sequence, necessitating the use of TCA. The WPRE sensor contained the TAA stop codon. Only when AA was converted to II (read as GG) was the translation of the downstream nano-luciferase activated. Of note, a high level of translational readthrough was observed in the sensor reporter, which was unexpected. As shown in Figure 5, the nano-luciferase-to-firefly luciferase ratio was approximately 0.05 for the SMN and GFP sensors and 0.4 for the WPRE sensor, which are not in line with previous reports that suggested that the termination efficiency of TAA is better than that of TAG [29,30]. For the WPRE sensor, although the activation of the nano-luciferase required the conversion of two adenosines to inosines, its response (SNR) was not inferior to that of the SMN and GFP sensors, suggesting that the editing efficiency for AA is similar to that for A. To reduce the background luciferase activity, two tandem stop codons were introduced into the sensor sequence to increase the efficiency of translation termination. Thus, SMN dsensor 1 containing a single stop codon was used as a control. Meanwhile, SMN dsensor 1 + 2 contained two consecutive stop codons, and SMN dsensor 1 + 3 contained two stop codons separated by a 15-nucleotide sequence. As shown in Figure 2d, the three sensors showed similar luciferase background activity, suggesting that there are other mechanisms responsible for the background activity of nano-luciferase. As expected, the A-to-G editing efficiency of SMN dsensor 1 was the highest. However, surprisingly, the A-to-G editing efficiency of SMN dsensor 1 + 2 was lower than that of dsensor 1 + 3. The reason for this phenomenon may be that the binding of the ADAR protein to one A shields another nearby A (not AA), thereby reducing its accessibility for editing. These findings indicate that the distance between two As has a significant impact on the process of coediting. Previous reports have shown that ADAR1 and ADAR2 overexpression can increase RNA editing efficiency [23,31]. A mutant ADAR2 (E488Q) has been shown to exhibit a higher RNA editing capacity [32,33]. In the current study, different ADARs, including ADAR1, ADAR2, and mutant ADAR2 (E488Q), were cotransfected. The results showed that overexpressed ADARs resulted in a higher luciferase background. However, no improvement in assay sensitivity and SNR was observed, suggesting that endogenous ADARs are sufficient for the mRNA reporter assay. Additional experiments are needed to verify whether other mechanisms besides ADAR-mediated RNA editing are involved in the activation of nano-luciferase, although it is clear from the specificity validation data that the activation is induced by a specific target sequence. Many attempts were made to optimize the assay, such as AAV receptor overexpression and cell pretreatment with hydroxyurea, but only prolonged incubation time could effectively increase the sensitivity and SNR of the assay.

After confirming the response of the sensor-based reporter to plasmids, other types of gene therapy vectors, including mRNA, lentivirus, and AAV, were analyzed. All these vectors exhibited a dose-dependent effect on specific sensor reporter cells. For AAV vectors, in particular, the sensor reporter assay could discriminate between different serotypes in terms of transduction efficiency, indicating that it can be employed as a potency assay for AAV products. Next, the suitability of the assay to determine the transcriptional activity of rAAV products was assessed. To verify the specificity of the sensors, AAV samples harboring different elements were evaluated using SMN, GFP, and WPRE sensor reporter 293T cells. The results revealed that each sensor responded only to the AAV sample containing its target sequence, suggesting high specificity. As a quantitative method, 4-PL parallel analysis is widely employed in cell-based potency assays [34,35,36]. The developed reporter assay fit the 4-PL model, with an R^2^ of >0.99. Considering that high AAV titer has a negative effect on the cell function, resulting in reduced firefly luciferase activity, which is also observed in lentivirus, the nano-luciferase-to-firefly luciferase ratio (reflecting the efficiency of RNA editing) was used as the response. F-tests revealed no significant nonlinearity or nonparallelism (*p* = 0.05), indicating that the 4-PL parallel analysis is suitable for the reporter assay. The precision, accuracy, and linearity of the reporter assay were not inferior to conventional cell-based bioassays [37,38,39,40]. Of note, the linear range of the assay was very wide, and test samples with lower activity (such as AAV2-GFP-WPRE sample 2) gave precise and accurate results. Overall, the developed reporter assay showed a similar dose–response relationship to the protein assay (GFP expression), suggesting that it can be used as an alternative method for the potency testing of gene therapy products. Furthermore, its high precision, accuracy, and linearity made it suitable for the batch release and stability analyses of gene therapy products. The development of such a reporter assay only requires the screening and design of a target-specific sensor sequence, followed by its substitution for the existing sensor sequence. This streamlined approach greatly simplifies the method development process compared with existing assays.

Limitations of this study and future technical developments: The present study is based on the 293T cell line, which is known for its high susceptibility to AAV infection and inherent expression of ADARs. For other cell types, including those derived from neural tissue, overexpressed ADARs may be essential to increase the sensitivity of the assay. Furthermore, the assay is designed to target mRNA, and although we have established a correlation between mRNA and protein expression, this relationship may not be consistent in all biological contexts. This limitation suggests that further validation of the assay’s performance in different cell types and under different conditions is required. Finally, the underlying mechanism of the assay should be investigated to verify whether there is another mechanism other than ADAR-mediated mRNA editing, which will be of great importance for further improvements in assay performance.

## 4. Materials and Methods

### 4.1. Materials

Plasmid pNLF1-C was purchased from Promega (Madison, WI, USA), and plasmid pAAV-GFP-WPRE and five AAV serotypes (AAV2-GFP, AAV5-GFP, AAV6-GFP, AAV8-GFP, and AAV9-GFP) were purchased from VectorBuilder (Shanghai, China). The AAV2-GFP-WPRE reference, sample 1, and sample 2 were derived from distinct production batches. Plasmids pAAV-SMN-GFP-WPRE and AAV2-SMN-GFP-WPRE were specially custom-made by VectorBuilder (Shanghai, China). The AAV2-SMN-GFP-WPRE reference and sample 1 also originated from different batches. The genomic titer of AAV was determined using inverted terminal repeat-targeting qPCR. Meanwhile, ADAR1 plasmid was custom-made by HEBIO (Shanghai, China), and ADAR2 and mutant ADAR2 (E488Q) plasmid were generously gifted by the laboratory of Dr. Yuan Peng-fei. Circle mRNA carrying GFP sequence was provided by CATUG Biotechnology (Shanghai, China), lentivirus harboring GFP and WPRE sequences was provided by OBiO Technology (Shanghai, China), and adenovirus carrying GFP sequence was purchased from VectorBuilder (Shanghai, China).

JetMESSENGER mRNA transfection reagent was obtained from Polyplus (Illkirch-Graffenstaden, France), while Fugene HD Transfection Reagent, Nano-Glo Live Cell Assay System, and Nano-Glo Dual-Luciferase Reporter Assay System were purchased from Promega (Madison, WI, USA). In addition, Opti-MEM was bought from GIBCO (Brooklyn, NY, USA), SpectraMax iD3 was sourced from Molecules Devices (San Jose, CA, USA), and Castor X1 High-throughput Smart Cell Analyzer was provided by Countstar (Shanghai, China). Finally, TaqMan Universal Master Mix II (2×), Absolute QTM DNA Digital PCR Master Mix (5×), and QuantStudioTM Absolute QTM MAP16 Digital PCR Kit (with MAP16 plates, gaskets, and Absolute Q Isolation Buffer) were purchased from Thermo Fisher Scientific (Waltham, MA, USA).

### 4.2. Construction of Sensor Plasmid

Firefly luciferase/nano-luciferase double-reporter sensor plasmid was constructed from pNLF1-C plasmid by inserting the tandem firefly luciferase sequence, T2A, sensor, and F2A to the upstream of nano-luciferase. Table 3 lists the sensor sequences and corresponding target sites.

### 4.3. Establishment of Stable-Transfected Sensor Cells

Sensor plasmids were transfected into 293 or 293T cells using Fugene HD Transfection Reagent according to the manufacturer’s instructions. The selection medium (Dulbecco’s modified Eagle’s medium supplemented with 10% fetal bovine serum and 100 µg/mL hygromycin B) was regularly replaced, and the cells were continuously cultured for 4 weeks. The response of the reporter cells was assessed using a plasmid carrying a specific sequence.

### 4.4. Transcriptional Activity Assays

293T cells transfected with the sensor plasmid were adjusted to a concentration of 7 × 10^5^ cells/mL, seeded into a clean-bottomed white 96-well plate at 100 μL per well, and cultured overnight. For plasmid assays, the plasmid sample was diluted to a concentration of 100 ng/μL in TE buffer and subjected to a twofold serial dilution. Next, 20 μL of each dilution was dispersed in 100 μL of Opti-MEM medium and thoroughly mixed with 6 μL of transfection reagent. The mixture was incubated at room temperature for 10 min, followed by its addition to each well (10 µL) and culture for 48–72 h. The luciferase activity assay was performed at 48 or 72 h using a Nano-Glo Live Cell Assay System according to the manufacturer’s instructions.

For mRNA assays, the sample was diluted to a concentration of 100 ng/μL in mRNA buffer, followed by fivefold serial dilution. Next, 20 μL of each dilution was dispersed in 100 μL mRNA buffer. To this solution, 6 μL transfection reagent was added and mixed thoroughly. The mixture was incubated for 10 min at room temperature, and 10 μL of this mixture was added to each well of the cell plate and cultured for 48–72 h. The luciferase activity assay was performed at 48 or 72 h using a Nano-Glo Live Cell Assay System.

For lentivirus assays, 20 μL of virus was added to 380 μL of Opti-MEM and serially diluted twofold. The cell supernatant was removed, and 50 μL of the diluted virus solution was added to the cell plate and cultured for 72 h. Then, 150 μL of complete medium was added to each well at 12–16 h, and luciferase activity assay was performed at 72 h using a Nano-Glo Dual-Luciferase Reporter Assay System.

For AAV assays, the virus was diluted 100-fold in Opti-MEM and serially diluted twofold. The cell supernatant was removed, and 50 µL of the diluted virus solution was added to each well and incubated for 72–120 h. Then, 150 µL of complete medium was added to each well at 12–16 h, and luciferase activity assay was performed at 72, 96, or 120 h using a Nano-Glo Dual-Luciferase Reporter Assay System.

### 4.5. AAV Genomic Titer Determination by dPCR

The genomic titer of AAV samples was determined using dPCR targeting the GFP sequence. The forward primer (5′-GCTGGAGTACAACTACAAC-3′), reverse primer (5′-TGGCGGATCTTGAAGTTC-3′), and probe (5′VIC-CTTGATGCCGTTCTTCTGCTTGT-MGB3′) were synthesized by Sangon (Shanghai, China). DNase I was used to digest any free nucleic acids in the sample at 37 °C for 30 min, followed by enzyme inactivation with 5 mM EDTA. Prior to the dPCR assay, the sample was diluted at least 1000-fold with 0.05% F68-TE buffer. The total volume for dPCR reaction was 10 μL, comprising 2 μL 5× Digital PCR Master Mix, 0.8 μL forward primer (10 μM), 0.8 μL reverse primer (10 μM), 0.4 μL probe (10 μM), 5 μL DNA template, and 1 μL PCR-grade water. Of the mixture, 9 μL was added to MAP16 plates, followed by sample analysis on a QuantStudio Absolute Q Digital PCR System (Thermo Fisher Scientific, Waltham, MA, USA). dPCR was performed in two stages: initial denaturation at 96 °C for 10 min and 50 cycles of denaturation and annealing at 96 °C for 15 s and 55 °C for 1 min, respectively.

### 4.6. Precision, Accuracy, and Linearity Assessment

To assess the precision of the assay, samples were analyzed five times, and the RSD of the relative potencies was calculated. In addition, to estimate the accuracy and linearity of the assay, the serial dilutions of AAV2-GFP or AAV2-SMN-GFP reference were evaluated. The potency of the initial dilution was set at 100% and that of the other dilutions was 80%, 60%, 40%, and 20%. The potencies of 80%, 60%, 40%, and 20% were calculated against the 100% sample using the 4-PL parallel logistic assay. The recovery of each dilution was calculated by dividing the measured potency by the expected potency. Linearity was analyzed using a linear equation, with y denoting measured values and x signifying expected values.

### 4.7. Statistical Analysis

4-PL curve fitting was performed using GraphPad Prism 9.5.0 software and relative potency calculations were performed using PLA3.0 software. The transcriptional activity of the reference was set to 100%, and the relative potency of the test samples was calculated against the reference sample using the 4-PL parallel assay. The nano-luciferase-to-firefly luciferase ratio was used as the response and the 1/dilution factor was considered the dose in the 4-PL parallel assay. F-tests (*p* = 0.05) were used for analyzing sample suitability, including the significance of nonlinearity and nonparallelism.

## 5. Conclusions

In this study, an RNA editing-based reporter assay, which exhibited its utility in evaluating the expression level of therapeutic genes in gene therapy products, was developed. This novel assay offers a robust and reliable platform for assessing the biological activity of gene therapy products, thereby effectively addressing the complexities and challenges associated with traditional potency assays. The high specificity, sensitivity, and broad dynamic range of the developed assay make it a powerful tool for quality control within the gene therapy industry. In addition, its innovative design offers a significant advantage over existing methods, ultimately helping streamline the process of evaluating gene expression and enhancing the accuracy of potency measurements.

## Figures and Tables

**Table 1:** Relative potency estimates of AAV samples.

Sample	Genomic Titer (vg/mL)	Sensor	Test 1 (%)	Test 2 (%)	Test 3 (%)	Test 4 (%)	Test 5 (%)	Mean (%)	RSD (%)
AAV2-GFP-WPRE reference	4.80 × 10^12^	GFP sensor	/	/	/	/	/	/	/
AAV2-GFP-WPRE sample 1	1.61 × 10^11^	GFP sensor	3.35	3.19	2.50	2.46	2.78	2.86	14.0
AAV2-GFP-WPRE sample 2	2.86 × 10^12^	GFP sensor	76.9	68.3	60.7	70.4	70.0	69.3	8.4
AAV2-SMN-GFP-WPRE reference	5.21 × 10^12^	SMN sensor 1	/	/	/	/	/	/	/
AAV2-SMN-GFP-WPRE sample 1	4.17 × 10^12^	SMN sensor 1	20.4	19.3	20.8	19.7	18.3	19.7	4.9

**Table 2:** Accuracy and linearity.

Expected Value (%)	AAV2-GFP		AAV2-SMN-GFP	
	Measured Value (%)	Recovery Rate (%)	Measured Value (%)	Recovery Rate (%)
100	100.0	/	100.0	/
80	74.2	92.7	74.0	92.5
60	56.6	94.3	57.7	96.2
40	41.3	103.4	42.5	106.3
20	22.5	112.3	22.5	112.3
Linear equation *	y = 0.974x		y = 0.9788x	
R^2^	0.9981		0.9979	

* x, the expected value; y, the measured value.

**Table 3:** Sensor sequences and target sites.

Sensor	Target Site	Sequence
GFP sensor	+587nt~+685nt of GFP	5′-cggcggcggtcacgaactccagcaggaccatgtgatcgcgcttctcgtTAG*ggtctttgctcagggcggactgggtgctcaggtagtggttgtcgggca-3′
WEPR sensor	+58nt~+156nt of WPRE	5′-gatttatacaaggaggagaaaatgaaagccatacgggaagcaatagcaTAAtacaaaggcattaaagcagcgtatccacatagcgtaaaaggagcaaca-3′
SMN sensor	+30nt~+128nt of SMN	5′-tatgcttttatcagtgctgtatcatcccaaatgtcagaatcatcgctcTAGcctgtgccgcgccggaacagcacggaatcctcctgctccgggacgccg-3′
SMN dsensor 1	+691nt~789nt of SMN	5′-catacttcccaaagcatcagcatcatcaagagaatctggacatatgggaggtggTAGgggaattattggtggtccagaaggaaatggaggcagccagca-3′
SMN dsensor 1 + 2	+691nt~789nt of SMN	5′-catacttcccaaagcatcagcatcatcaagagaatctggacatatgggaggTAGTAGgggaattattggtggtccagaaggaaatggaggcagccagca-3′
SMN dsensor 1 + 3	+691nt~789nt of SMN	5′-catacttcccaaagcatcagcatcatcaagagaatcTAGacatatgggaggtggTAGgggaattattggtggtccagaaggaaatggaggcagccagca-3′

* The letters highlighted in red are the termination codons.

## Data Availability

Data are contained within the article. Further inquiries can be directed to the corresponding authors.
